# Associations Between Changes in Levels of Phosphorylated Tau and Severity of Cognitive Impairment in Early Alzheimer Disease

**DOI:** 10.1212/WNL.0000000000213676

**Published:** 2025-05-15

**Authors:** Fernando Gonzalez-Ortiz, Bjørn-Eivind Kirsebom, Yara Yakoub, Julia K. Gundersen, Lene Pålhaugen, Knut Waterloo, Per Selnes, Jonas Alexander Jarholm, Berglind Gísladóttir, Arvid Rongve, Ragnhild Eide Skogseth, Geir Bråthen, Dag Aarsland, Michael Turton, Peter Harrison, Henrik Zetterberg, Sylvia Villeneuve, Tormod Fladby, Kaj Blennow

**Affiliations:** 1 Inst. of Neuroscience and Physiology, University of Gothenburg, Mölndal, Sweden;; 2 Clinical Neurochemistry Lab, Sahlgrenska University Hospital, Mölndal, Sweden;; 3 Department of Neurology, University Hospital of North Norway, Tromsø, Norway;; 4 Department of Psychology, Faculty of Health Sciences, The Arctic University of Norway, Tromsø, Norway;; 5 Department of Neurology, Akershus University Hospital, Lørenskog, Norway;; 6 Douglas Mental Health University Institute, Centre for Studies on the Prevention of Alzheimer's Disease (StoP-AD), Montreal, Quebec, Canada;; 7 Division of Physiology, Institute of Basic Medical Sciences, University of Oslo, Norway;; 8 University of Oslo, Institute for Clinical Medicine, Campus Ahus;; 9 Department of Neuropsychology, Haugesund Hospital, Norway;; 10 Department of Clinical Medicine (K1), University of Bergen, Norway;; 11 Department of Geriatric Medicine, Haraldsplass Deaconess Hospital, Bergen, Norway;; 12 Department of Clinical Sciences, Faculty of Medicine, University of Bergen, Norway;; 13 Department of Neurology and Clinical Neurophysiology, University Hospital of Trondheim, Norway;; 14 Department of Neuromedicine and Movement Science, Faculty of Medicine and Health Sciences, Norwegian University of Science and Technology, Norway;; 15 Centre for Age-Related Diseases. Stavanger University Hospital Stavanger, Norway;; 16 Department of Old Age Psychiatry. Institute of Psychiatry, Psychology and Neuroscience King's College London, United Kingdom;; 17 Bioventix Plc, Surrey, United Kingdom;; 18 Department of Neurodegenerative Disease, UCL Institute of Neurology, London, United Kingdom;; 19 UK Dementia Research Institute at UCL, London, United Kingdom;; 20 Hong Kong Center for Neurodegenerative Diseases, China;; 21 Wisconsin Alzheimer's Disease Research Center, University of Wisconsin School of Medicine and Public Health, University of Wisconsin-Madison, WI;; 22 Paris Brain Institute, ICM, Pitié-Salpêtrière Hospital, Sorbonne University, France; and; 23 Neurodegenerative Disorder Research Center, Division of Life Sciences and Medicine, and Department of Neurology, Institute on Aging and Brain Disorders, University of Science and Technology of China and First Affiliated Hospital of USTC, Hefei, P.R. China.

## Abstract

**Background and Objectives:**

Aligning biomarker evidence with clinical presentation in early Alzheimer disease (AD) is essential for improving diagnosis, prognosis, and interventions. This study evaluates the relationship between cognitive impairment, future decline, and phosphorylated tau levels in plasma and CSF in predementia AD.

**Methods:**

This longitudinal observational study included predementia cases and controls from 2 independent cohorts: the Norwegian Dementia Disease Initiation (DDI) and Canadian Pre-Symptomatic Evaluation of Experimental or Novel Treatments for Alzheimer's Disease (PREVENT-AD). In DDI, cognitively normal (CN) and mild cognitive impairment (MCI) cases were classified using CSF Aβ42/40 ratio (A) and p-tau181 (T), whereas classification in PREVENT-AD (A) was based on amyloid PET scans. In DDI, we assessed CSF-plasma correlations for p-tau181, p-tau217, and p-tau231. Diagnostic accuracies were evaluated through receiver operating characteristic analyses. Linear mixed models evaluated p-tau associations with future memory decline. Between-group differences in plasma p-tau217 were assessed in both cohorts.

**Results:**

In DDI (n = 431), participants were classified as CN A−/T− (n = 169), A+/T− (CN = 26, MCI = 24), A+/T+ (CN = 40, MCI = 105), and A−/T+ (CN = 34, MCI = 33), with a mean age of 64.1 years and 55.9% female. In PREVENT-AD (n = 190), participants were categorized as CN A− (n = 118), CN A+ (n = 49), and MCI A+ (n = 21), with a mean age of 67.8 years and 72.6% female. In DDI, plasma p-tau217 showed high accuracy in identifying A+ participants (areas under the curve [AUC]: 0.85) and a moderate correlation with CSF p-tau217 (rho = 0.65, *p* < 0.001). Diagnostic accuracy of plasma p-tau217 was greater in MCI A+ (AUC: 0.89) than in CN A+ (AUC: 0.79, *p* < 0.05) and in A+/T+ (AUC: 0.88) vs A+/T− (AUC: 0.78, *p* < 0.05). p-Tau181 and p-tau231 had weaker CSF-plasma correlations (rho = 0.47 and rho = 0.32, *p* < 0.001) and were less associated with cognitive status in A+ individuals. Higher plasma p-tau217 in A+ MCI vs A+ CN individuals (*p* < 0.001) was confirmed in PREVENT-AD. All CSF p-tau markers, but only plasma p-tau217, were associated with future memory decline (β = 0.05, *p* < 0.05).

**Discussion:**

Our findings suggest that, unlike p-tau181 and p-tau231, plasma p-tau217 consistently aligns with cognitive status in A+ individuals and better reflects CSF biomarker abnormalities, reducing discrepancies between clinical and biochemical findings. Its association with baseline and future memory decline highlights its diagnostic and prognostic value, particularly when CSF analysis or PET is unavailable.

## Introduction

Alzheimer disease (AD) represents the most common cause of dementia in older populations.^1^ However, despite its high prevalence, a clinical diagnosis of AD remains challenging because of its insidious symptomatology, particularly in early stages of the disease.^2-4^ While definite diagnosis is based on the neuropathologic evidence of the main AD hallmarks, ß-amyloid plaques and tau tangles in the brain,^1,5^ the use of neuroimaging (PET) and CSF biomarkers that corroborate the presence of these hallmarks has proven to be valuable to support clinical diagnosis.^1,6,7^ These pathologic changes are often detected in the brain well before any noticeable cognitive change, reflecting the preclinical or prodromal stages,^2,8^ which may explain why biologically defined AD is more prevalent than clinically defined AD.^9^

Classification or staging of AD pathology can be assessed through biological and cognitive measurements. Biological classification relies on objective markers identified through imaging or CSF analysis (e.g., amyloid PET and CSF amyloid 42/40 ratio). These biomarkers provide valuable insights into the disease at the molecular level and categorize patients according to the presence of amyloid and tau pathology.^10^ On the contrary, clinical classification of AD involves the assessment of cognitive and functional decline.^11^

In recent years, sensitive techniques for assessing biomarkers in plasma have emerged as accessible methods for detecting and potentially predicting early AD changes.^6,12^ Plasma p-tau markers such as p-tau181, p-tau217, and p-tau231 have shown promising performance at identifying early AD pathology.^7,8,12^ Among these markers, plasma p-tau217 has shown a superior accuracy in early stages of the disease continuum, serving as a possible first-in-line diagnostic test.^13,14^

Although plasma biomarkers hold great promise for early AD diagnosis and prognosis,^15^ they do not consistently align with the clinical presentation.^16^ Some individuals with substantial biomarker evidence of AD pathology may exhibit mild clinical symptoms, whereas others with fewer biomarker abnormalities may experience more severe cognitive deficits.^3,16^ Moreover, the temporal evolution of AD biomarkers is not linearly correlated with cognitive decline.^17,18^ The rate of change in biomarker levels may vary greatly between individuals, and clinical symptoms may worsen rapidly or slowly, making it challenging to predict disease progression accurately.^17^ The reasons for this are not completely understood but may relate to the temporal disconnect between biomarker evidence of pathology buildup and neuronal network breakdown/loss of brain resilience.^19^ However, aligning biomarker evidence with clinical presentation is essential for identifying patients suitable for current anti-amyloid treatments.^20^ While CSF p-tau181 is clinically approved for AD diagnostics, both plasma and CSF p-tau217 have been proposed as more sensitive markers for pathologic disease burden.^21^

In this study, we investigate changes in p-tau biomarkers, with a focus on plasma, in relation to clinical presentation in predementia AD. We also explore their prognostic capabilities by examining associations with cognitive status and future cognitive deterioration in early AD. In addition, we assess the influence of renal function on these markers by examining their associations with glomerular filtration rate (GFR). Finally, we compare the positive predictive values (PPVs) and negative predictive values (NPVs) of each plasma p-tau marker to determine their precision and appropriateness of use at different clinical stages of predementia AD.

## Methods

### Standard Protocol Approvals, Registrations, and Patient Consents

This study was performed with pertinent ethical approvals. Dementia Disease Initiation (DDI) has been approved by the Regional Committees for Medical and Health Research Ethics in Norway (REK: 2013/150).^22^ All participants provided written informed consent before participating in the study.^22^ For Pre-Symptomatic Evaluation of Experimental or Novel Treatments for Alzheimer's Disease (PREVENT-AD), written informed consent was obtained from all participants and all research procedures were approved by the Institutional Review Board at McGill University. Detailed description of inclusion and exclusion criteria for the DDI and PREVENT-AD studies has previously been outlined.^22,23^

### DDI

The DDI study is a Norwegian multicenter cohort study recruiting participants from 6 major hospitals across Norway (University Hospital of North Norway, Tromsø; St. Olavs University Hospital, Trondheim; Akershus University Hospital, Lørenskog; Haraldsplass Deaconal Hospital, Bergen; Stavanger University Hospital, Stavanger; and Haugesund Hospital, Haugesund).^22^ The study had been ongoing since 2013, and ethical approvals are in place until 2030. Inclusion criteria are age between 40 and 80 years and native language of Norwegian, Swedish, or Danish. The exclusion criteria are intellectual disability or other developmental disorders, brain trauma, stroke, dementia, severe psychiatric conditions, or severe somatic disease that might influence cognitive functions. The DDI protocol comprises extensive medical history, neurologic and neuropsychological examinations, lumbar puncture, blood draw, and brain magnetic resonance imaging (MRI). Participants were primarily recruited from the memory clinics of their respective hospitals. In addition, participants were also recruited through advertisements in local news media targeting individuals with a family history of dementia or those experiencing cognitive symptoms. Participants are invited for reassessments approximately every 2 years after initial baseline assessment following the protocol outlined above until withdrawal from the study, or until end point—defined as reaching the dementia stage. Participants with cognitive symptoms are categorized as subjective cognitive decline (SCD) or mild cognitive impairment (MCI) staged according to published criteria^24,25^ regardless of biomarker status (CSF or MRI). Cognitive impairment is defined as performance 1.5 SD below the normative mean in one or more cognitive domains, including delayed memory recall (Consortium to Establish a Registry for Alzheimer's Disease [CERAD] word list test),^26^ executive function (Trail Making Test Part B),^27^ language/verbal fluency (Controlled Oral Word Association Test),^28^ and visuoperceptual ability (Visual Object and Space Perception Battery silhouettes).^29^ Cognitive normalcy was defined as performance above this threshold across all assessed domains. However, as previously applied in the DDI cohort,^30^ here we use an actuarial definition of cognitive normalcy and MCI based on neuropsychological test battery performance as outlined above, independent of SCD experience (details in eMethods).

### PREVENT-AD

PREVENT-AD is a longitudinal observational study consisting of 385 initially cognitively unimpaired older adults with a parental or multiple-sibling history of AD dementia.^23^ PREVENT-AD is the primary clinical research initiative of the Centre for Studies on Prevention of Alzheimer's Disease (StoP-AD Centre). The program aims to investigate memory and brain changes in healthy individuals older than 55 years. While some in this age group may start experiencing noticeable memory issues, many others could have hidden brain changes that signal the early stages of the disease long before symptoms appear. Participants in the PREVENT-AD study are aged 60 years or older on entry, or 55 years or older if within 15 years of their relative's symptom onset. All participants underwent Clinical Dementia Rating test and Montreal Cognitive Assessment on enrollment.^23^ These individuals underwent neuropsychological evaluation using Repeatable Battery for the Assessment of Neuropsychological Status, MRI, and blood draw for routine laboratory results. A subsample of participants underwent positron emission tomography (PET) scans of Aβ pathology.

### CSF and Blood Proteomics

In the DDI cohort, CSF Aβ1-42 and CSF Aβ1-40 concentrations were measured using the QuickPlex SQ 120 system from Meso Scale Discovery (MSD, MD). The Aβ42/40 ratio was used to determine Aβ plaque pathology (cutoff ≤0.077).^31^ CSF samples included before October 2020 were analyzed using commercial enzyme-linked immunosorbent assays from Innotest, Fujirebio, Ghent, Belgium, based on monoclonal antibodies to determine CSF phosphorylated tau (p-tau181) concentration. Owing to a change in laboratory equipment, CSF samples included after October 2020 were analyzed using Elecsys p-tau181 kits (n = 421 were determined with Innotest [>65 pg/mL]; n = 10 were determined with Elecsys [>19 pg/mL]).^30^ The PREVENT-AD cohort did not include CSF samples, and the presence of AD pathology was assessed with Aβ PET.

All the p-tau markers in DDI and PREVENT-AD were measured on the Simoa HD-X platform with one in 30 dilution factor in CSF (only available in the DDI cohort) and 2-fold factor in plasma. p-Tau181, p-tau231, and p-tau217 were measured according to published methods.^7,32,33^ Signal variations within and between analytical runs were assessed using 3 internal quality control samples at the beginning and the end of each run.

### Aβ-PET

In the PREVENT-AD cohort, Aβ-[18F NAV4694] PET scans were performed at the McConnell Brain Imaging Centre at the Montreal Neurological Institute. Aβ-PET scans were obtained 40–70 minutes after injection (220 MBq). The images were reconstructed using a three-dimensional (3D) ordinary Poisson ordered subset expectation maximum algorithm with 10 iterations and 16 subsets.^34^ The data were preprocessed using our in-house protocol that is available at github.com/villeneuvelab/vlpp. Standardized uptake value ratios (SUVRs) were calculated for each region of the Desikan-Killiany atlas by dividing the tracer uptake in the cerebellar gray matter for Aβ-PET scans. A global amyloid index SUVR threshold of 1.27, equivalent to centiloid = 18, was selected for Aβ-PET positivity.

### Study Design

We selected all cases and controls at baseline from the DDI cohort according to the following criteria: (1) cognitively normal participants (CN) (controls or those with SCD, after neuropsychological test results) with normal CSF Aβ42/40 ratio and normal p-tau181 biomarkers (CN A−/T−); (2) CN or MCI participants with pathologic Aβ42/40 ratio, but normal p-tau181 (A+/T); (3) those with pathologic Aβ42/40 ratio and p-tau181 (A+/T+); and (4) those with normal Aβ42/40 ratio but pathologic p-tau181 (A−/T+). Verbal episodic memory decline is acknowledged as one of the earliest clinical features of AD.^35^ Because our study concerns cases without dementia, in the presumed preclinical (CN) or prodromal (MCI) stages of AD, we opted to only assess longitudinal associations between plasma/CSF p-tau markers and future verbal episodic memory decline (CERAD word list delayed memory recall). In the PREVENT-AD cohort, we included all available cases and controls with plasma p-tau217 data available in whom amyloid status was determined using Aβ PET. Participants in the PREVENT-AD cohort were classified as A− CN, A+ CN, or A+ MCI.

### Statistics

Between-group differences were assessed using 1-way analysis of variance for A/T groups in the DDI cohort and for CN/MCI (A−/A+) groups in both DDI and PREVENT-AD. Age and log-transformed plasma p-tau were analyzed in both cohorts while log-transformed CSF p-tau and GFR were assessed only in DDI. Post hoc comparisons were adjusted using the false discovery rate. Categorical variables (sex, diagnoses, and *APOE* genotype) were assessed with χ² tests in both cohorts. A subanalysis of the biomarker differences between CN and MCI cases within the DDI pathologic A/T groups was conducted with independent-sample *t* tests. Spearman rho correlations were performed between CSF and plasma p-tau epitopes both in the complete DDI sample and within A/T groups. Fisher z-transformation was used to compare the correlation coefficients. Relative mean changes in both CSF and plasma biomarkers were computed for CN and MCI pathologic A/T groups with the mean biomarker concentrations for the CN A−/T− group (CN Aβ− for between cohort comparisons) as the reference ($\frac{CN\;group-A/T\;\text{group}}{CN\;group)}$. Receiver operating characteristic analyses were performed for cognitive (CN A− vs CN A+ and MCI A+) and biological (CN A−/T− vs A+/T− and A+/T+) status and compared using the Delong test. Cutoffs for each model were generated using the Youden index, and NPVs and PPVs were computed accordingly. In DDI, linear mixed models were fitted to assess associations between baseline p-tau epitopes in CSF/plasma and future memory decline (CERAD word list recall subtest^36^) for A− (A−/T− and A−/T−) and A+ (A+/T− and A+/T−) separately (eTables 1 and 2). Spearman rho correlations between the plasma biomarkers and GFR were performed in the complete DDI sample (additional details in eMethods).

### Data Sharing

Anonymized aggregated-level data will be shared by request from a qualified academic investigator for the sole purpose of replicating procedures and results presented in the article, and as long as data transfer is in agreement with European Union legislation on the general data protection regulation and decisions by the ethical review boards in charge of each of the cohorts used for this study.

## Results

### Sample Characteristics and Demographics in Our Cohorts

A total of 431 participants from the DDI cohort were included, categorized based on CSF AD biomarkers: A+/T− (n = 50; CN: n = 26, MCI: n = 24), A+/T+ (n = 145; CN: n = 40, MCI: n = 105), and A−/T+ (n = 67; CN: n = 34, MCI: n = 33). In addition, 169 CN A−/T− individuals were included as a reference group. The average age across groups ranged from 60.04 to 67.86 years, with groups exhibiting pathologic biomarkers being older (by 4.47–7.86 years on average) than the CN A−/T− group. While the A+/T− group had a higher proportion of women (74%) compared with other groups (50%–57%), this difference was not statistically significant. As expected, APOE-ε4 carriers were more prevalent in the A+ groups (74%–77%) compared with the A− groups (32%–44%) (details in the Table). In the PREVENT-AD cohort, 190 participants with available Aβ-PET scans were included: 119 CN A−, 49 CN A+, and 21 MCI A+. The groups had similar average ages, ranging from 67.33 to 70.04 years (eTable 3). Compared with the DDI cohort, a higher proportion of women were represented in all groups (68%–82%). However, no significant differences in gender distribution were observed between groups. As expected, APOE-ε4 carriers were more prevalent in the A+ groups (54%–56%) compared with the A− group (27%).

**Table:** Between-Group Comparisons of Demographics, *APOE-ε4* Carrier Status, Diagnoses, and Plasma and CSF p-Tau Markers in the Dementia Disease Initiation (DDI) Cohort

	A/T groups (n)					Post hoc comparisons (p)		
	CN A−/T−169	A+/T−50	A+/T+145	A−/T+67	*F*/*χ*^*2*^/*η*^*2*^ (*p*)	A+/T−vs A+/T+	A+/T−vs A−/T+	A+/T+ vs A−/T+
Age								
Mean (SD)	60.04 (9.13)	66.66*** (7.52)	67.86*** (7.85)	64.51*** (9.66)	*F* = 23.13, *η*^*2*^ = 0.14 (<0.001)	n.s^b^	n.s^b^	<0.05^b^
Female								
n (%)	94 (56)	36 (72)	73 (50)	38 (57)	*χ*^*2*^ = 7.10, (0.068)	^b^	^b^	^b^
APOE-ε4 carrier status n (%) (n)	53 (32) (164)	35 (74) (47)	106 (77) (138)	27 (44) (62)	*χ*^*2*^ = 70.54, (<0.001)	^b^	^b^	^b^
Diagnoses					*χ*^*2*^ = 182.94, (<0.001)			
CN (n %)	169 (100)	26 (52)	40 (28)	34 (51)				
MCI (n %)	0 (0)	24 (48)	105 (72)	33 (49)				
Plasma p-tau181^a^ Mean (SD) (n)	9.92 (7.03) (168)	13.00*** (6.41)	15.71*** (6.84) (143)	11.62* (6.73) (66)	*F* = 34.07, *η*^*2*^ = 0.19 (<0.001)	<0.01^b^	n.s^b^	<0.001^b^
Plasma p-tau217^a^ Mean (SD) (n)	1.70 (0.70) (161)	2.68*** (1.12) (49)	3.54*** (1.53) (144)	1.99* (1.04) (66)	*F* = 76.01, *η*^*2*^ = 0.35 (<0.001)	<0.001^b^	<0.001^b^	<0.001^b^
Plasma p-tau231^a^ Mean (SD) (n)	5.28 (3.86)	6.30 (4.54) (49)	7.91*** (5.07)	6.31 (4.11) (65)	*F* = 9.41, *η*^*2*^ = 0.06 (<0.001)	n.s^b^	n.s^b^	n.s^b^
CSF p-tau181^a^ Mean (SD) (n)	107.89 (78.16) (167)	206.26 (122.78)	505.84*** (450.57) (141)	168.48*** (104.70) (64)	*F* = 142.5, *η*^*2*^ = 0.51 (<0.001)	<0.001^b^	n.s^b^	<0.001^b^
CSF p-tau217^a^ Mean (SD) (n)	46.23 (24.11)	106.25*** (43.63)	226.27*** (72.20) (144)	73.49*** (32.92) (64)	*F* = 395.2, *η*^*2*^ = 0.74 (<0.001)	<0.001^a^	<0.001^a^	<0.001^b^
CSF p-tau231^a^ Mean (SD) (n)	284.46 (134.35) (167)	506.63*** (168.08)	1,126.52*** (562.90) (141)	411.93*** (129.22) (64)	*F* = 300.8, *η*^*2*^ = 0.68 (<0.001)	<0.001^b^	<0.01^b^	<0.001^b^
GFR mean (SD) (n)	79.49 (14.76) (110)	79.96 (12.33) (45)	81.04 (14.00) (128)	75.46 (15.03) (52)	*F* = 1.94, (0.123)	^c^	^c^	^c^

Abbreviations: % = percentage; A+/− = positive or negative CSF marker for Aß plaques; APOE = apolipoprotein E; CN = cognitively normal; F = F statistic; GFR = glomerular filtration rate; MCI = mild cognitive impairment; n = number of cases; T+/− = positive or negative marker for CSF p-tau181; vs = versus; η^2^ = eta-squared; χ^2^ = chi-square statistic.

a Measured in pg/mL.

b Analysis of variance post hoc (false discovery rate adjustment).

c No post hoc comparisons performed.

*<0.05,** <0.01, ***<0.001 (compared with the CN A−/T− group).

### Agreements Between CSF and Plasma p-Tau Biomarkers

**Figure 1:**
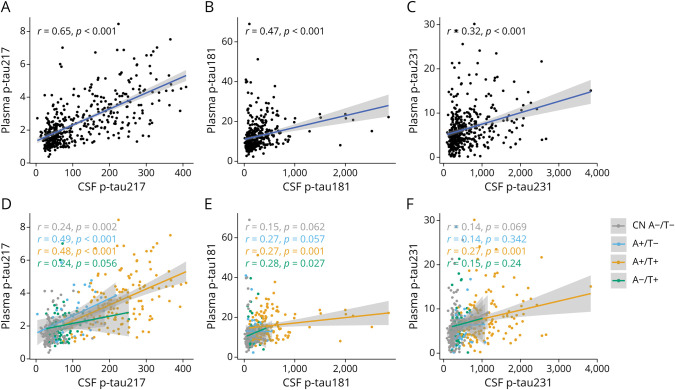
Agreements Between CSF and Plasma p-Tau Biomarkers in the DDI Cohort Scatterplots illustrating the Spearman rho correlations between plasma and CSF p-tau markers. (A–C) Correlations of plasma p-tau217, p-tau181, and p-tau231 with their corresponding CSF markers. (D–F) CSF-plasma correlations of p-tau217, p-tau181, and p-tau231 in the different A/T groups. DDI = Dementia Disease Initiation.

In the DDI cohort, we observed a moderate correlation between CSF and plasma p-tau217 (rho = 0.65, *p* < 0.001, Figure 1A) and markedly weaker correlations for p-tau181 (z = 3.83, *p* < 0.001, rho = 0.47, *p* < 0.001, Figure 1B) and p-tau231 (z = 6.38, *p* < 0.001, rho = 0.31, *p* < 0.001, Figure 1C). Split by A/T groups, CSF-plasma p-tau217 correlations (Figure 1D) were similar in both A+/T− (rho = 0.49, *p* < 0.001) and A+/T+ (rho = 0.48, *p* < 0.001) groups. Weaker CSF-plasma correlations were seen in the CN A−/T− and A−/T+ groups (both rho = 0.24, *p* < 0.01; *p* = 0.056). For both p-tau181 (Figure 1E) and p-tau231 (Figure 1F), CSF-plasma correlations were weaker in all groups compared with p-tau217 (rho between 0.14 and 0.28). In this study, both p-tau181 and p-tau231 showed more robust correlations in the A+/T+ group (both rho = 0.27, *p* < 0.001). Notably, the similarity in correlations of CSF-plasma p-tau217 in A+/T− and A+/T+ groups highlights its ability to reliably capture CSF pathologic changes, even in early stages of AD. By contrast, the weaker and more variable correlations for p-tau181 and p-tau231 suggest that these markers may be less sensitive to CSF-related biomarker abnormalities, limiting their utility in aligning plasma measurements with underlying pathology.

### Diagnostic Performance Based on Cognitive Status vs Biological Status

**Figure 2:**
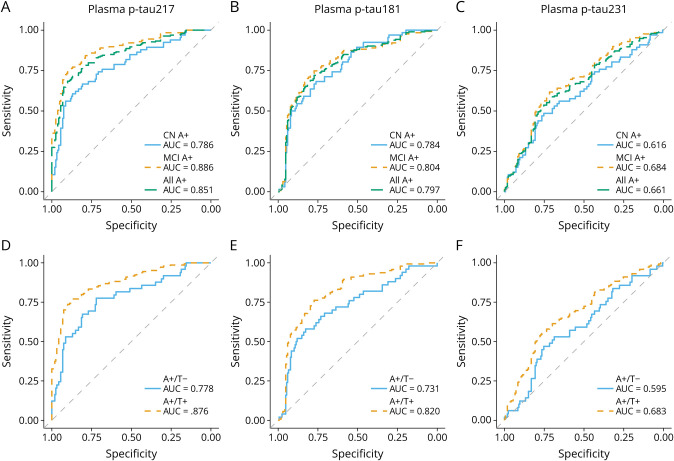
Diagnostic Accuracy of Plasma p-Tau Markers in the DDI Cohort Receiver operating characteristic (ROC) curves and corresponding areas under the curve (AUCs) showing the discriminative ability of the different plasma p-tau biomarkers. (A–C) ROC curves and AUCs of plasma p-tau217, p-tau181, and p-tau231 identifying Aβ+ individuals based on their cognitive status. (D–F) ROC curves and AUCs of plasma p-tau217, p-tau181, and p-tau231 identifying Aβ+ individuals according to their A/T profile in CSF. DDI = Dementia Disease Initiation; ROC = receiver operating characteristic.

In the DDI cohort, plasma p-tau217 demonstrated the highest area under the curve to distinguish A+ from A− individuals regardless of the cognitive status (0.850), followed by p-tau181 (0.797) and p-tau231 (0.661). However, only p-tau217 showed a significant increase in accuracy differentiating MCI A+ (0.886) from CN A+ (0.786, *p* < 0.05) as well as A+/T− (0.778) from A+/T+ (0.876) (*p* < 0.05) (details in Figure 2 and eTables 4 and 5). Plasma p-tau217 had PPVs and NPVs above 0.800 for MCI A+ and A+/T+ vs A−/T− controls but showed lower PPVs for CN A+ and A+/T− (0.656 and 0.458, respectively) (details in eTable 6 and eFigure 1). In CSF, p-tau217 also showed the highest accuracy for identifying A+ individuals (0.973), followed by p-tau231 (0.961) and p-tau181 (0.906). In this study, all epitopes had higher accuracies for identifying A+/T+ compared with A+/T− (between *p* < 0.01 and *p* < 0.001). However, only CSF p-tau231 differentiated between CN (0.942) and MCI A+ (0.970) (*p* < 0.05) (details in eFigure 2 and eTables 5 and 6).

### Between-Group Differences in CSF and Plasma p-Tau and Associations With Memory Decline

**Figure 3:**
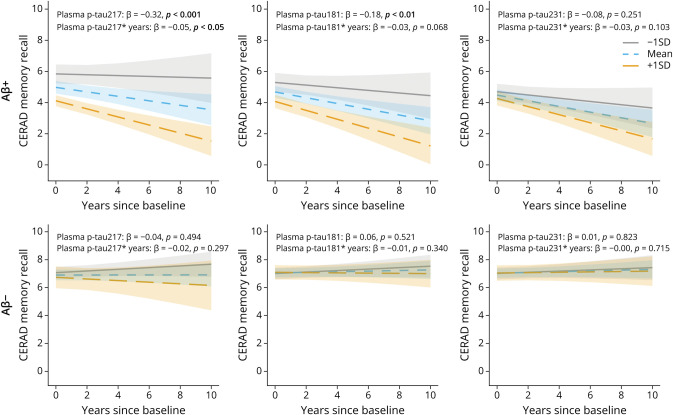
Baseline and Longitudinal Associations of Plasma p-Tau Markers With the Consortium to Establish a Registry for Alzheimer's Disease (CERAD) Memory Recall Test in the DDI Cohort (A–C) Baseline and longitudinal associations of plasma p-tau217, p-tau181, and p-tau231 with the CERAD memory recall test in Aβ+ individuals. (D–F) Baseline and longitudinal associations of plasma p-tau217, p-tau181, and p-tau231 with the CERAD memory recall test in Aβ- individuals. The lines display associations between the biomarker at −1SD (gray), mean (blue), and +1SD (orange) and the dependent variable at baseline and over time. DDI = Dementia Disease Initiation.

In CSF, p-tau217 and p-tau231 were higher in all pathologic A/T groups (all *p* < 0.001) while p-tau181 was higher only in the A+/T+ and A−/T+ groups (both *p* < 0.001; details in the Table). When splitting by cognitive status, only plasma p-tau217 was higher in MCI A+/T+ compared with CN A+/T+ (*p* < 0.05), whereas only CSF p-tau217 and CSF p-tau231 had higher concentrations in MCI A+/T+ compared with CN A+/T+ (*p* < 0.001) (eTable 7). Relative mean change (with CN A−/T− as the reference) in biomarker concentrations corresponded to the between-group differences outlined above, notably demonstrating generally a higher relative mean change for both plasma and CSF p-tau217 in the A+ groups compared with the other p-tau epitopes and also a higher relative mean change for plasma p-tau 217 in MCI compared with CN A+/T+ (eFigure 3). Moreover, plasma p-tau217, but not p-tau181 or p-tau231, showed significant associations with both baseline (β = −0.32, *p* < 0.001) and future (β = −0.05, *p* < 0.05) verbal memory decline in A+ but not in A− participants (Figure 3 and eTable 1). In CSF, all p-tau epitopes associated with memory impairment and decline in A+ participants; however, CSF p-tau217 showed the strongest associations over time (β = −0.06, *p* < 0.05) and the highest NPV and PPV (eTable 8). p-Tau181 was the only CSF p-tau marker to associate with future memory decline in the Aβ− participants (β = −0.04, *p* < 0.01) (eFigure 4 and eTable 2 for details).

### Plasma p-Tau Biomarkers According to Cognitive Status

**Figure 4:**
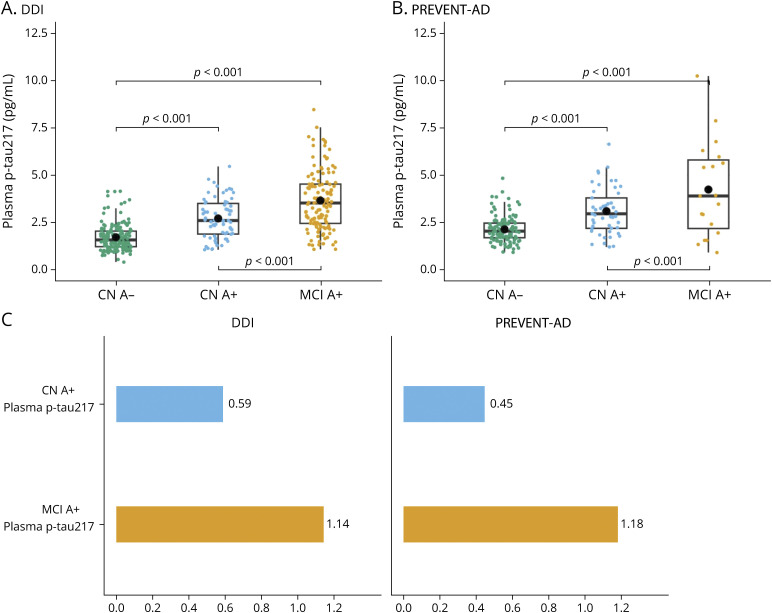
Plasma p-Tau217 Concentrations and Relative Mean Change Increases According to Cognition in the DDI and PREVENT-AD Cohorts (A and B) Boxplots showing concentrations of plasma p-tau217 (pg/mL) in CN Aβ−, CN Aβ+, and MCI Aβ+ individuals in DDI and PREVENT-AD. The brackets show statistically significant differences between the groups (FDR-adjusted *p* values). (C) The bar graphs illustrate the relative mean change increases of plasma p-tau217 in Aβ+ CN and Aβ+ MCI participants compared with CN Aβ− in the DDI and PREVENT-AD cohorts. CN = cognitively normal; DDI = Dementia Disease Initiation; PREVENT-AD = Pre-Symptomatic Evaluation of Experimental or Novel Treatments for Alzheimer's Disease; FDR = false discovery rate; MCI = mild cognitive impairment.

In both DDI and PREVENT-AD cohorts, we compared plasma p-tau217 concentrations between CN A+ (DDI: n = 66; PREVENT-AD: n = 49) and MCI A+ (DDI: n = 127; PREVENT-AD: n = 21) groups relative to CN A− (DDI: n = 161; PREVENT-AD: n = 118). In DDI, A± was defined based on pathologic or normal CSF Aβ42/40 ratios while in PREVENT-AD, it was determined by pathologic or normal amyloid-PET scans. In both cohorts, we found significantly higher p-tau217 concentrations in the MCI A+ participants compared with CN A+ (both cohorts *p* < 0.001, Figure 4, A and B). In DDI, we also compared plasma p-tau181 and p-tau231 between groups but found no difference in concentrations between CN A+ and MCI A+ participants (both n.s., eFigure 5). Moreover, relative mean changes in plasma p-tau217 for the A+ groups (compared with CN A−) were remarkably similar in both cohorts (Figure 4C), thus replicating between independent cohorts that plasma p-tau217 is sensitive to cognitive severity in predementia AD regardless of the method used to determine Aβ positivity. Results of plasma p-tau181 and p-tau231 in PREVENT-AD have previously been reported.^34^

### Biomarker Correlations With Glomerular Function

DDI had a subset of participants with available GFR data (335/431, 77.7%). In this study, plasma p-tau217 showed the weakest association with GFR (rho = −14, *p* < 0.05), followed by p-tau181 (rho = −17, *p* < 0.01) and p-tau231 (rho = −22, *p* < 0.001). As expected, no associations between GFR and CSF p-tau epitopes were found. No differences in GFR between the A/T groups were observed (Table).

## Discussion

While all p-tau markers in CSF demonstrated excellent diagnostic and prognostic accuracy, in plasma, only p-tau217 exhibited diagnostic and prognostic performance comparable with CSF p-tau markers. The superior performance of plasma p-tau217 over plasma p-tau181 and plasma p-tau231 at detecting early AD biochemical signatures and its sensitivity to capture cognitive changes might be attributed to its unique properties observed in in vitro models, such as promoting synaptic decline and the formation of tau-tau interactions at the expense of tau binding to microtubules.^37^

In the DDI cohort, when we divided A+, determined by the CSF *Aβ42/40 ratio*, according to their cognitive status (CN A+ and MCI A+), plasma p-tau217 showed significant elevation in the MCI A+ group compared with CN A+ group while plasma p-tau181 and p-tau231 did not show any difference between these groups. We replicated the plasma p-tau217 results in the PREVENT-AD cohort, in which AD pathology was determined by Aβ-PET, where higher levels of plasma p-tau217 were associated with worse cognitive performance in A+ participants. Moreover, relative mean changes in biomarker concentrations compared with the control group were remarkably similar in the CN A+ and MCI A+ groups for both cohorts (Figure 4).

When dividing A+ participants in the DDI cohort according to their CSF profiles (Aβ42/40 ratio and p-tau181, A+/T− and A+/T+), we observed that in the A+/T− group, none of the CSF or plasma biomarkers could differentiate between CN individuals and those with MCI. On the contrary, all the CSF p-tau markers and plasma p-tau217, but not plasma p-tau181 or p-tau231, were capable of distinguishing between CN and MCI in the A+/T+ group. These findings suggest that while cognitive deterioration might affect the levels of plasma p-tau217 in A+ individuals, it is likely that the joint pathology (A+/T+) is the main driver of significant increases in plasma p-tau217. It is important to note, however, that in our study, T+ was determined using clinically approved CSF p-tau181 assays, rather than the more appropriate CSF p-tau217 assay. Relatedly, we also demonstrate that relative mean changes for p-tau217 are greater in CSF than in plasma (eFigure 3), with diagnostic concordance to CSF Aβ42/40-defined Aβ pathology being correspondingly higher in CSF. Furthermore, CSF p-tau217 outperforms CSF p-tau181 in this context,^21^ underscoring its robustness as a marker of core AD pathology when measured in CSF compared with plasma. Although plasma p-tau217 has shown diagnostic accuracy comparable with CSF-based Aβ42/40 or p-tau181/Aβ42,^38^ we propose that CSF p-tau217 likely represents a more appropriate benchmark for assessing the diagnostic performance of plasma p-tau biomarkers. Moreover, evidence suggests that elevated CSF p-tau181 may not reliably reflect tau-tangle pathology as a state marker because it identifies A+/T− subtypes with significant clinical impairment and progression.^39^ While other p-tau epitopes have yet to be evaluated in this context, CSF p-tau217 shows a stronger association with tangle burden, as measured by in vivo tau-PET imaging,^21^ highlighting its potential as a more clinically relevant marker of tau-tangle pathology in AD.

When comparing the performance of CSF vs plasma p-tau217 in the DDI cohort, we observed that even with less pronounced elevation in biomarker concentrations compared with controls, plasma p-tau217 accurately identified *A+* participants. Furthermore, when we assessed the PPV and NPV of plasma p-tau markers, plasma p-tau217 showed a superior performance identifying true-positive (TP) and true-negative (TN) participants when compared with p-tau181 and p-tau231 (eFigure 1 and eTable 6). However, all the plasma p-tau markers showed a poor performance identifying TP participants in the CN A+ and in the A+/T− groups. Our results suggest that the optimal diagnostic performance of plasma p-tau217, based on PPV and NPV, was achieved in the A+ MCI participants and in those with an A+/T+ profile in CSF regardless of cognitive status. These findings are particularly relevant for real-world settings, where relying solely on a single measurement of plasma p-tau217 might lead to misdiagnosis, if this is not taken in consideration. Lower PPVs for preclinical AD cases have been reported previously^13^ and are believed to result from the lower pretest probability of amyloid pathology in CN individuals.^40^ However, as we and others show,^15,41^ higher concentrations of plasma p-tau217 correspond to degree of clinical impairment and future decline and may thus track pathologic and clinical severity.^42^ This is important because not all CN A+ participants will develop MCI or dementia in their lifetimes.^43^ Moreover, CN participants with higher concentrations of plasma p-tau217 may be at greater risk, and 2 cutoff approaches^44^ aimed at reducing the diagnostic mismatch with CSF or amyloid-PET may also serve to capture those with higher likelihood of clinical progression. This may be of particular importance for identifying patients eligible for current anti-amyloid treatments.^20^

Of interest, in Aβ+ cases, the association with future verbal memory decline was similar for plasma and CSF p-tau217. By contrast, CSF p-tau181 emerged as the only marker associated with memory decline in Aβ- cases (Figure 3 and eFigure 4). This group included CN and MCI individuals with CSF A−/T+ profiles, referred to as suspected nonamyloid pathology (SNAP). SNAP cases are clinically and neuropathologically heterogeneous, generally carrying a lower risk of clinical progression.^45^ However, our findings suggest that elevated CSF p-tau181 may be associated with an increased risk of memory decline in this group, whereas p-tau217 does not. Together, these findings underscore the specificity of p-tau217 as a core marker of AD pathology and highlight its prognostic value in both CSF and plasma. Furthermore, in the DDI cohort, we observed that plasma p-tau217 levels were less affected by kidney function when compared with p-tau181 and p-tau231, suggesting a higher robustness. These findings, in addition to the CSF-plasma correlations observed in the DDI cohort, might suggest that, while in CSF, the 3 p-tau markers are all sensitive for detecting AD-related pathology, in blood, potential peripheral contribution of p-tau181 and p-tau231 could affect the diagnostic and prognostic performance of these markers while plasma p-tau217 seems to be less affected by peripheral factors and a more accurate reflection of AD pathology.

Levels of plasma p-tau217 align consistently with biological and clinical changes observed in AD providing valuable insights into the disease's progression, even in its early stages, namely preclinical and prodromal AD. While p-tau181 and p-tau231 have been valuable in AD research, our study underscores the unique diagnostic and prognostic potential of plasma p-tau217 in tracking cognitive changes. Moreover, our findings address the potential limitations of using plasma p-tau217 very early in the clinical trajectory of AD while also acknowledging that, owing to its minimally invasive nature and accessibility, plasma p-tau217 makes an excellent alternative for screening and routine clinical assessments when CSF analysis or PET is not available. However, it is crucial to interpret plasma p-tau217 levels within the appropriate clinical context to ensure accurate disease management. Integrating plasma p-tau217 into clinical practice holds promise not only for improving AD diagnosis but also to facilitate early interventions in patients at risk of cognitive decline.

The main strengths of our study include the use of 2 well-characterized, independent cohorts with extensive biomarker and cognitive assessments, allowing for robust comparisons and validation of findings. However, some limitations should be noted. We focused solely on assessing p-tau associations with verbal episodic memory decline because it is widely recognized as one of the earliest and most prominent clinical features of early AD.^35^ However, memory decline alone does not fully capture the broader cognitive and functional decline that occurs throughout the disease course, which we acknowledge as a limitation of our study. In addition, the lack of longitudinal blood sampling limits our ability to assess dynamic changes in plasma biomarkers over time. Finally, we acknowledge the low racial and ethnical diversity of our population.

## Glossary

AD: Alzheimer disease
AUC: areas under the curve
CERAD: Consortium to Establish a Registry for Alzheimer's Disease
CN: cognitively normal
DDI: Dementia Disease Initiation
GFR: glomerular filtration rate
MCI: mild cognitive impairment
NPV: negative predictive value
PPV: positive predictive value
PREVENT-AD: Pre-Symptomatic Evaluation of Experimental or Novel Treatments for Alzheimer's Disease
SCD: subjective cognitive decline
SNAP: suspected nonamyloid pathology
SUVR: standardized uptake value ratio
TP: true positive
